# Percutaneous Coronary Intervention Enhances Accelerative Wave Intensity in Coronary Arteries

**DOI:** 10.1371/journal.pone.0142998

**Published:** 2015-12-11

**Authors:** Om Narayan, Michael C. H. Leung, Dennis T. L. Wong, Ian T. Meredith, James D. Cameron

**Affiliations:** Monash Cardiovascular Research Centre, MonashHeart and School of Clinical Sciences at Monash Health, Monash University, Melbourne, Australia; University Hospital Medical Centre, GERMANY

## Abstract

**Background:**

The systolic forward travelling compression wave (sFCW) and diastolic backward travelling decompression waves (dBEW) predominantly accelerate coronary blood flow. The effect of a coronary stenosis on the intensity of these waves in the distal vessel is unknown. We investigated the relationship between established physiological indices of hyperemic coronary flow and the intensity of the two major accelerative coronary waves identified by Coronary Wave Intensity analysis (CWIA).

**Methodology / Principal Findings:**

Simultaneous intracoronary pressure and velocity measurement was performed during adenosine induced hyperemia in 17 patients with pressure / Doppler flow wires positioned distal to the target lesion. CWI profiles were generated from this data. Fractional Flow Reserve (FFR) and Coronary Flow Velocity Reserve (CFVR) were calculated concurrently. The intensity of the dBEW was significantly correlated with FFR (R = -0.70, P = 0.003) and CFVR (R = -0.73, P = 0.001). The intensity of the sFCW was also significantly correlated with baseline FFR (R = 0.71, p = 0.002) and CFVR (R = 0.59, P = 0.01). Stenting of the target lesion resulted in a median 178% (interquartile range 55–280%) (P<0.0001) increase in sFCW intensity and a median 117% (interquartile range 27–509%) (P = 0.001) increase in dBEW intensity. The increase in accelerative wave intensity following PCI was proportionate to the baseline FFR and CFVR, such that stenting of lesions associated with the greatest flow limitation (lowest FFR and CFVR) resulted in the largest increases in wave intensity.

**Conclusions:**

Increasing ischemia severity is associated with proportionate reductions in cumulative intensity of both major accelerative coronary waves. Impaired diastolic microvascular decompression may represent a novel, important pathophysiologic mechanism driving the reduction in coronary blood flow in the setting of an epicardial stenosis.

## Introduction

Despite recognizing that hyperemic coronary flow is reduced distal to a functionally significant coronary stenosis, an understanding of the precise pathophysiologic mechanisms underlying this abnormality is lacking. Recent evidence suggests that the suction effect generated by myocardial microvascular recoil in early diastole is the principal force driving coronary blood flow (CBF) [1]. The precise role of this suction effect in the setting of an epicardial stenosis remains unknown.

Physiological indices such as Fractional Flow Reserve (FFR) and Coronary Flow Velocity Reserve (CFVR) enable quantification of coronary flow impairment but fail to provide insights into the associated alteration of hemodynamic forces leading to this defect [2–4]. FFR is a well-validated measure of the trans-stenotic pressure gradient and functional significance of an epicardial coronary stenosis[5, 6]. CFVR and CFR are also well established as indices reflecting both epicardial and microvascular contributions to coronary flow restriction. All three indices are highly dependent on the microvascular response to vasodilating agents (adenosine and papaverine). The trans-stenotic pressure drop generated during hyperemia is reduced in the setting of a submaximal microvascular vasodilatory response, resulting in a correspondingly higher measured FFR [7]. Conversely, measured CFVR is typically lower in the presence of microvascular vasodilatory impairment [8]. As both indices represent averages of either pressure or flow velocity across several cardiac cycles, neither is capable of providing insights into the hemodynamic perturbations giving rise to the flow abnormality within a cardiac cycle, between systole and diastole.

**Fig 1:**
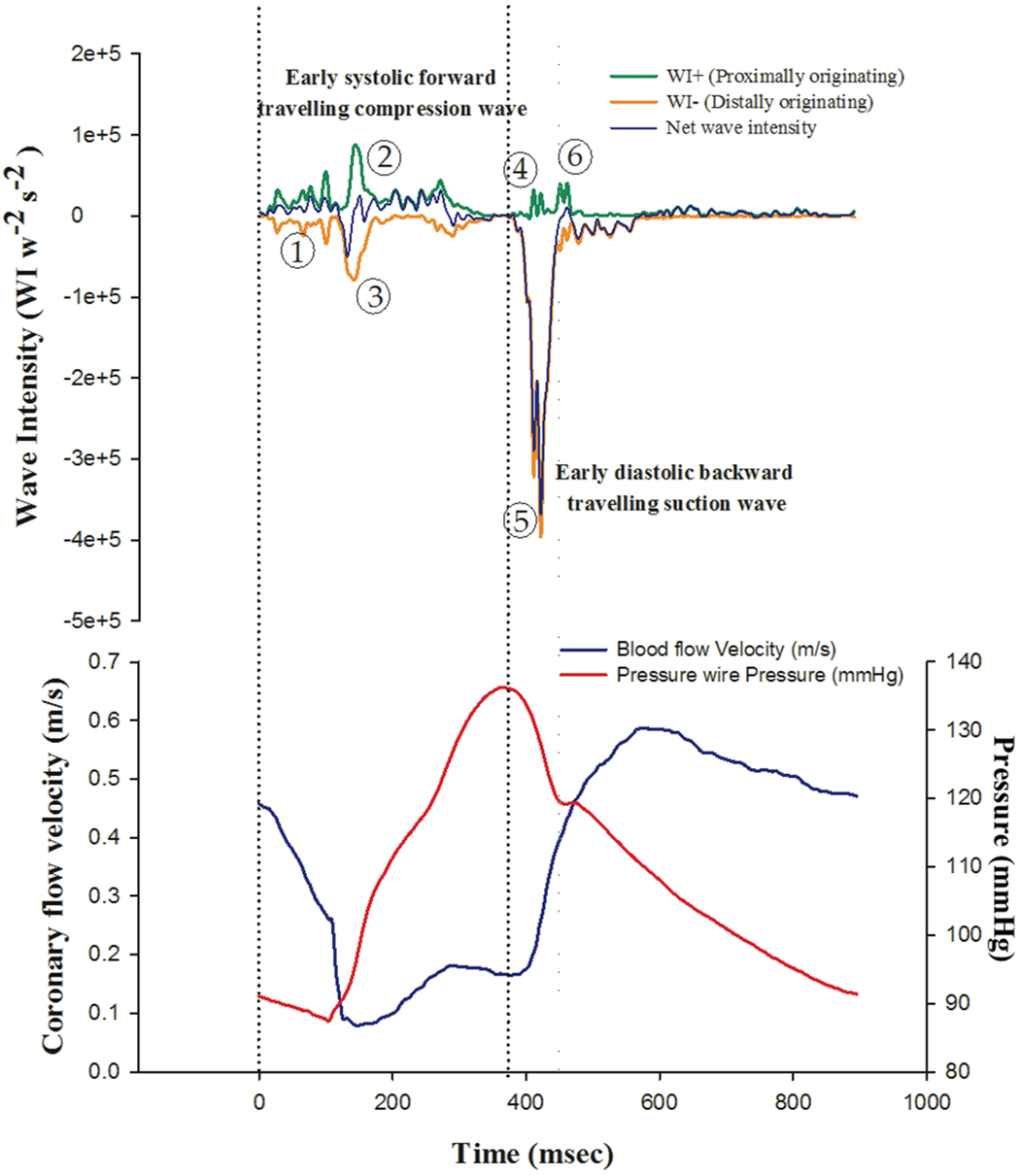
Origins and effects of waves within the coronary circulation. A representative example of coronary pressure and flow velocity with calculated net wave intensity and separated wave intensity profiles measured over a single cardiac cycle is shown. Measurements were taken in the distal vessel, following stenting of the target stenosis during resting conditions. 6 major waves have been identified within the coronary circulation. These waves are hypothesized to explain coronary pressure and flow velocity over a complete cardiac cycle. Waves are numbered according to their sequence of arrival during the cardiac cycle (see Table 1). The early systolic forward travelling compression wave (sFCW) and early diastolic backward travelling decompression wave (dBEW) have been proposed as the primary accelerative forces acting on coronary flow. FFR, Fractional Flow reserve; CFVR, Coronary Flow Velocity Reserve; sFCW, systolic Forward travelling Compression Wave; dBEW, diastolic Backward travelling Expansion Wave.

Coronary Wave Intensity Analysis (CWIA) was developed to characterize the magnitude and duration of the proximally and distally originating forces generating CBF over the cardiac cycle [9]. CWIA quantifies the transmission of energy within the circulation by ascribing the phasic changes in pressure and velocity to multiple travelling wavefronts [10]. These wavefronts summate to create waves that have the effect of either accelerating or decelerating coronary artery blood flow, depending on their direction (antegrade or retrograde) and type (compression or expansion) (Table 1 and Fig 1) [9]. CWIA has demonstrated the presence of two major waves that predominantly account for acceleration of CBF–a systolic forward travelling compression wave (sFCW) and a diastolic backward travelling decompression or “suction” wave (dBEW) [1]. The intensity of the diastolic backward travelling suction wave is attenuated in the setting of myocardial hypertrophy secondary to hypertension and with pacing in severe aortic stenosis [1, 11]. However no previous studies have assessed the relationship between the coronary wave intensity profile and established physiological measures of CBF (FFR and CFVR). The effects of percutaneous coronary intervention with stenting (PCI) on the coronary wave intensity profile also remain unknown.

**Table 1:** Identified waves within the epicardial coronary circulation. Proposed origins and effect on CBF of the 6 major coronary waves. Waves are presented in sequence of arrival over the cardiac cycle. Waves have been numbered to assist identification of waves in Fig 1.

Wave designation	Pressure	Velocity	Proposed wave origin	Effect on antegrade blood flow
1. Early Systolic Backward travelling Compression Wave	**↑**	↓	Myocardial systolic contraction leading to compression of cardiac microvessels	↓
2. Dominant Systolic Forward travelling Compression Wave (sFCW)	**↑**	↓	Left ventricular ejection	**↑**
3. Late Systolic Backward travelling Compression Wave	**↑**	↓	Reflection of forward travelling pushing wave from sites of impedance mismatch and systolic myocardial microvascular compression	↓
4. Early Diastolic Forward travelling Expansion Wave	↓	↓	Deceleration of ventricular ejection due to the initiation of myocardial relaxation	↓
5. Dominant early Diastolic Backward travelling Expansion Wave (dBEW)	↓	**↑**	Release of myocardial microvascular compression with a rapid reduction in coronary resistance	**↑**
6. Late diastolic forward travelling pushing wave	**↑**	**↑**	Aortic valve closure	**↑**

We hypothesized that the amplitude of both major accelerative coronary waves would be reduced in proportion to the functional significance of an epicardial stenosis (as measured by FFR and CFVR) and that relief of obstruction with PCI would result in an increase in wave intensity amplitude. We also postulated that the increase in accelerative wave intensity following PCI would be related to the severity of flow limitation at baseline.

## Methods

### Patient Population

Seventeen consecutive patients with stable angina pectoris undergoing clinically indicated elective PCI were prospectively enrolled between October 2002 and February 2004. Subjects were selected based on the presence of a single coronary lesion in an otherwise angiographically normal vessel. Exclusion criteria included significant left main coronary artery disease, triple vessel disease, prior coronary artery bypass graft surgery, a recent (<6 week) history of myocardial infarction, significant valvular heart disease, atrial fibrillation or conduction disturbances. Additionally, patients with known infarction in the territory subtended by the target vessel and bifurcational stenoses were excluded. All patients gave informed written consent, which conformed to the Declaration of Helsinki and the study protocol was approved by the Southern Health Human Research Ethics Committee.

### Cardiac Catheterization Procedure

All oral medications were continued until the cardiac catheterization with the exception of beta-blockers, which were withheld from 48hrs prior to the procedure. The procedure was performed transfemorally with a 6Fr-guiding catheter. Unfractionated heparin at a dose of 80 IU/kg was administered following sheath insertion. Intracoronary nitrogycerine (100mcg) was administered following the insertion of Doppler velocity and pressure guidewires before and after PCI to reduce the effect of coronary vasospasm on coronary hemodynamic measurements. Orthogonal angiographic projections of the target stenosis were analysed using proprietary software (Toshiba, Otawara, Japan) to determine percentage diameter stenosis.

### Coronary Hemodynamic measurements

Aortic pressure was obtained from a 6F-guiding catheter positioned at the ostium of the study artery. A 0.014-inch Doppler guidewire with a 12 MHz piezoelectric ultrasound transducer (FloWire^®^, Volcano Corp., Rancho Cordova, CA, USA), was advanced into the study artery through the guiding catheter and positioned at a minimum of 10 mm distal to the target stenosis. All measurements were performed in an angiographically normal vessel segment (<20% stenosis severity). A 0.014-inch pressure guidewire (Pressure Wire^™^, RADI Medical Systems, Uppsala, Sweden) was calibrated with the pressure console (Pressure Wire^™^ Interface) and with aortic pressure immediately proximal to the ostium of the study vessel. The pressure guidewire was then advanced until the transducer was adjacent to the Doppler guidewire, distal to the target stenosis. Both wires were manipulated until a stable, optimal signal was obtained.

Before PCI, the hemodynamic significance of the target lesion was established with measurement of the FFR and CFVR, during hyperemia induced with intracoronary boluses of adenosine. A dose of 24μg of intracoronary adenosine was used for the left coronary system and 18 μg for the right coronary artery [12, 13].

Simultaneous aortic pressure, intracoronary pressure, instantaneous peak velocity and ECG data were continuously acquired at a 200Hz sampling frequency, digitized (MacLab/8s System, ADInstruments, Castle hill, NSW, Australia) and analysed off-line. To determine the difference in signal processing times, we positioned both pressure and Doppler flow transducers adjacent to each other in a fluid filled tube, injected a bolus of saline into the tube and measured the time delay between the pressure and flow velocity signals. After 20 injections, there was an average time lag of 8.3 ms with the pressure signal preceding the Doppler velocity signal. The velocity signal was therefore advanced by 2 sample points (10 ms). Pre-PCI, all signals were acquired during resting conditions and subsequently during hyperemia with the guidewires positioned distal to the stenosis. After successful PCI, care was taken to precisely reposition the pressure and flow velocity guidewires at the same pre-PCI location (confirmed by comparison to the initial angiographic images) and all measurements, (including FFR and CFVR) were repeated. Given the sensitivity of CFVR and CFR estimation to changes in prevailing hemodynamic state, care was taken to perform all measurements during periods of heart rate and blood pressure stability. Left ventricular function was estimated with contrast left ventriculography performed prior to positioning of the guidewires. The incidence of peri-procedural infarction was estimated with the measurement of serum troponin I (TnI) drawn both prior to and following the procedure.

### Data Analysis

Data analysis was performed using customised software (MATLAB^™^, MathWorks, Inc, Natick, Mass. USA). An ensemble averaged pressure and velocity waveform was generated from 10 sequential waveforms for each set of measurements (pre PCI and post PCI) during resting conditions and following administration of intracoronary adenosine. An example of a wave intensity profile generated from a single pressure and velocity waveform (obtained over a single beat) is shown in Fig 1. Wave intensity analysis was performed with these ensemble averaged pressure and flow velocity waveforms. The concept of unseparated or “net” wave intensity refers to the sum of simultaneously occurring forward and backward travelling waves and hence indicates whether forward or backward travelling waves predominate at any given moment [14]. Net wave intensity is defined as the product of the incremental change in blood pressure and velocity over a small time interval[14]. Consequently net wave intensity provides an indication of the overall direction of wave transmission, it fails to resolve simultaneously occurring forward and backward travelling waves, which are often large and physiologically relevant.

In order to separate the net wave intensity profile into forward and backward travelling components, it is necessary to determine wave speed in the coronary artery. We utilized the single point wave speed method to estimate coronary wave speed [15], which has been shown to closely approximate coronary wave speed when measured during resting conditions [16]. This has been defined by Davies and colleagues [15] as:

$$SP_{c}\;=\;\frac{1}{\rho }\sqrt{\frac{\Sigma \text{d}P_{d}^{2}}{\Sigma \text{d}U^{2}}}$$

where dP_d_ and d*U* are the distal pressure and velocity differences, respectively, between successive sampling points (separated by a 5 millisecond delay) and ρ is the density of blood in kg/m^3^ (considered to be 1050 kg/m^3^). This technique avoids the need for dual pressure sensing guidewires and has been shown to closely approximate coronary wave speed when measured during resting conditions[16]. Wave intensity profiles were generated using the following equations [9, 10, 15]:

$$WI_{+}\;=\;\frac{1}{4\rho c}{\left(\frac{dP}{dt}+\rho c\frac{dU}{dt}\right)}^{2}$$

$$WI_{-}\;=\;-\frac{1}{4\rho c}{\left(\frac{dP}{dt}-\rho c\frac{dU}{dt}\right)}^{2}$$

where WI_+_ represents waves originating proximally (forward travelling) and WI_-_ indicates waves originating distally (within the coronary microvasculature) and propagating proximally, P represents distal coronary pressure measured in Pascals, U represents flow velocity measured in m/s, ρ indicates the density of blood (1050 kg/m^3^) and c is the estimated single-point coronary wave speed (SPc) in m/s. The onset and offset of each wave was identified through superposition of the pressure and flow velocity waveforms with the wave intensity profile. Cumulative wave intensity was estimated by integration of the area under individual waves [1].

Whereas the estimation of SPc enables separation of the coronary wave intensity profile into forward and backward travelling components, the validity of this approach in the setting of an epicardial stenosis is uncertain. Therefore we also calculated unseparated or “net” coronary wave intensity profiles (where estimation of SPc is not required) [17, 18]. Whilst individual waves cannot be resolved with this approach, the overall net direction and amplitude of wave transmission can be estimated. Cumulative intensities calculated for the two major accelerative waves were compared with the peak intensities of net forward travelling compression and backward travelling decompression, to determine the applicability of the wave separation approach to stenosed coronary arteries.

### Statistical analysis

Data are expressed as means ± SD or medians and interquartile ranges as appropriate. Spearman’s rank coefficient test and simple linear regression were used to determine the correlation between non-normally distributed continuous variables. Statistical analysis was performed using GraphPad Prism version 5 (GraphPad Software, Inc). Statistical significance was assumed at p < 0.05.

## Results

### Baseline patient characteristics

The baseline characteristics of the 17 patients enrolled in this study were typical of those undergoing elective PCI at our institution (Table 2). The left anterior descending artery was the most commonly identified target vessel, representing 47.1% of cases, followed by the right coronary artery with 29.1% of cases. Hemodynamic data was successfully acquired in all patients (Table 3). The median pre-PCI FFR was 0.59 (interquartile range 0.39–0.80) versus 0.92 (interquartile range 0.88–0.98) post PCI (P<0.001). Left ventricular function as assessed by quantitative contrast left ventriculography was normal (50–69%) in 14 / 17 of included patients, mildly abnormal in 2 / 17 (40–49%) and moderately abnormal in 1 / 17 (30–39%). Additional information regarding baseline angiographic, haemodynamic and procedural parameters are presented in Figs A and B in S1 File.

**Table 2:** Baseline Demographics.

	Number (%) n = 17
Age (mean ± SD)	64±10.8
Gender (male, %)	8 (47)
Hypertension	11 (65)
Diabetes	2 (12)
Hypercholesterolemia	17 (100)
Smoking	11 (65)
Body Mass Index (mean ± SD)	26.8 (5.1)
Previous MI	9 (53)
**Target artery**	
LAD	8 (47)
LCx	4 (24)
RCA	5 (29)
**Medications**	
Beta blockers	12 (71)
Statins	15 (88)
Nitrates	6 (35)
ACEi	8 (47)
Calcium Channel blockers	4 (24)
Aspirin	16 (94)
Clopidogrel	7 (41)
**Left Ventricular Function**	
Normal	14 (82)
Mild dysfunction	2 (12)
Moderate dysfunction	1 (6)

**Table 3:** Baseline Hemodynamic Parameters.

	Mean ± SD (n = 17)
HR (beats per minute) (mean±SD)	57.4 ± 8.7
Systolic blood pressure (mmHg)	126.0 ± 21.9
Diastolic blood pressure (mmHg)	67.0 ± 9.0
Pulse pressure (mmHg)	59.0 ± 17.0
Systemic arterial compliance (units)	0.18 ± 0.1
Pulse wave velocity (m/s)	7.21 ± 2.9
Rate Pressure Product (mmHg.bpm)	7036 ± 1750.5
Fractional Flow Reserve	0.58 ± 0.2
Coronary Flow reserve	1.72 ± 0.6

### Wave speed changes with PCI and hyperemia

Consistent with previous studies [17], pre-PCI estimated SPc decreased significantly following intracoronary adenosine administration (mean baseline SPc 37.0 ± 3.4 m/s, mean post IC adenosine 20.4 ± 2.0 m/s, P<0.001). A decrease in SPc following PCI was evident when both pre-PCI and post PCI measurements were performed during resting conditions and adenosine induced hyperemia (mean pre PCI 37.0 ± 3.4 m/s, mean post PCI 23.5 ± 2.4 m/s, P < 0.003 during resting conditions and 20.4 ± 2.0 m/s vs. 11.9 ± 1.2 P = 0.001 during adenosine induced hyperemia). Following PCI, the administration of adenosine resulted in decreased SPc (mean SPc 23.5 ± 2.4 m/s during resting conditions, mean post IC adenosine 11.9 ± 11.2 m/s, P<0.001). Only wave speed estimates obtained during resting conditions were used in subsequent calculations of cumulative wave intensity.

### Comparison of cumulative accelerative wave intensity with Net (unseparated) wave intensity

Cumulative wave intensities obtained following wave separation with the single-point wave speed method were compared with peak net wave intensities (independent of wave speed). The peak intensity of net forward travelling compression was strongly correlated with the cumulative intensity of the sFCW measured both pre and post PCI (R = 0.89, P<0.0001 and R = 0.86, P<0.0001 respectively). Similarly, the peak intensity of net backward travelling decompression was strongly correlated with the cumulative intensity of the dBEW measured both pre and post PCI (R = 0.77, P = 0.001 and R = 0.83, P<0.0001).

### Accelerative wave intensity following PCI

Significant increases in cumulative wave intensity following PCI were noted for the sFCW (6.65 vs. 16.25 10^3^ W m^-2^ s^-1^ P<0.0001), the systolic backward travelling compression wave (2.61 vs. 4.40 10^3^ W m^-2^ s^-1^ P = 0.031) and the dBEW (11.93 vs. 28.10 10^3^ W m^-2^ s^-1^ P = 0.007) (Table 4). Conversely, the cumulative intensities of the early systolic backward travelling compression wave (4.20 vs. 5.56 10^3^ W m^-2^ s^-1^ P = 0.263), early diastolic forward travelling decompression wave (2.31 vs. 6.11 10^3^ W m^-2^ s^-1^ P = 0.064) and late diastolic forward travelling compression wave (3.37 vs. 3.50 10^3^ W m^-2^ s^-1^ P = 0.927) did not increase significantly following PCI (Table 4).

**Table 4:** Changes in Cumulative Wave intensity following PCI during adenosine induced hyperemia. Cumulative wave intensity profiles were generated using pressure and flow velocity signals acquired during hyperemia and SPc measured during resting conditions. The Wilcoxon Rank sum statistic was applied to estimate statistical significance.

	Cumulative Wave intensity pre-PCI (10^3^ W m^-2^ s^-1^) (SEM)	Cumulative Wave Intensity post-PCI (10^3^ W m^-2^ s^-1^) (SEM)	P value
1. Early Systolic Backward travelling Compression Wave	4.20 (0.88)	5.56 (0.75)	0.26
2. Dominant Systolic Forward travelling Compression Wave (sFCW)	6.65 (1.13)	16.25 (2.16)	**<0.0001**
3. Late Systolic Backward travelling Compression Wave	2.61 (0.42)	4.40 (0.63)	0.031
4. Early Diastolic Forward travelling Expansion Wave	2.31 (0.61)	6.11 (2.11)	0.064
5. Dominant early Diastolic Backward travelling Expansion Wave (dBEW)	11.93 (2.07)	28.06 (4.26)	**0.0007**
6. Late diastolic forward travelling pushing wave	3.37 (0.96)	3.50 (0.92)	0.927

### Accelerative wave intensity and baseline FFR / CFVR

**Fig 2:**
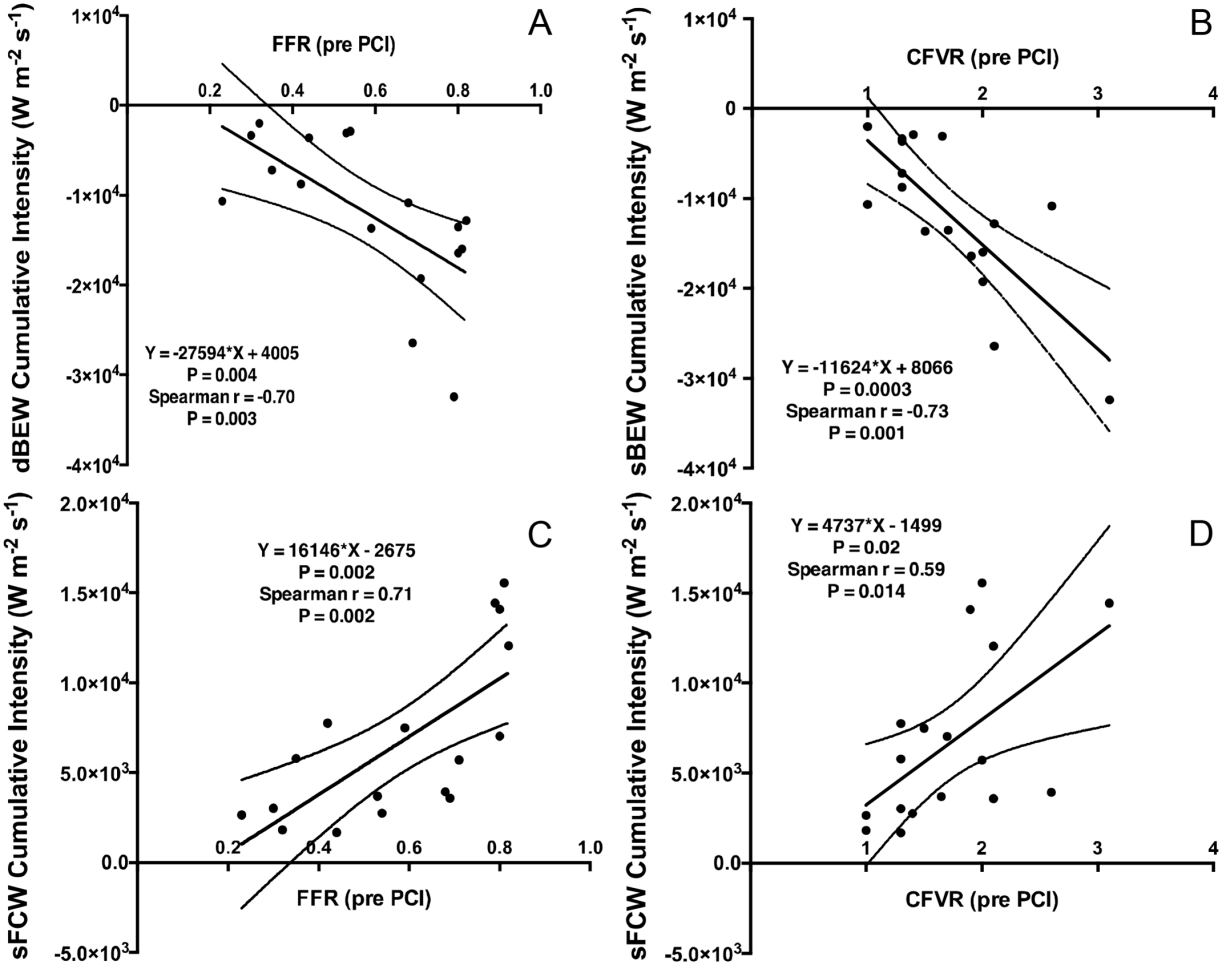
Relationship of Pre-PCI FFR / CFVR with Accelerative Wave intensity. **(A)** Fractional Flow Reserve and cumulative intensity of the dBEW, **(B)** Coronary Flow Velocity Reserve and cumulative intensity of the dBEW, **(C)** Fractional Flow Reserve and cumulative intensity of sFCW. **(D)** Coronary Flow Velocity Reserve and cumulative intensity of the sFCW. Equations for the regression lines and 95% confidence intervals are shown. All measurements were taken distal to the stenosis, prior to PCI, during adenosine-induced hyperemia. FFR, Fractional Flow reserve; CFVR, Coronary Flow Velocity Reserve; sFCW, systolic Forward travelling Compression Wave; dBEW, diastolic Backward travelling Expansion Wave.

Cumulative dBEW intensity was correlated with baseline FFR and CFVR (R = -0.70, P = 0.003 and R = -0.73 P = 0.001 respectively) (Table 5, Fig 2A and 2B). Cumulative sFCW intensity was also correlated with baseline FFR and CFVR (R = 0.71, P = 0.002 and R = 0.59, P = 0.014) (Fig 2C and 2D). Correlations with FFR and CFVR were not observed for the cumulative intensities of any other coronary wave. Additional information regarding correlations between FFR, CFVR, dBEW intensity, sFCW intensity and baseline angiographic, haemodynamic and patient parameters are presented in Tables C and D in S1 File).

**Table 5:** Correlations between cumulative accelerative wave intensity and FFR / CFVR. Correlations between FFR and CFVR measured distal to the lesion pre PCI with cumulative intensities of the two major accelerative coronary waves and the percentage change in cumulative intensity of these waves with PCI. The Spearman correlation coefficient was applied to estimate statistical significance.

	FFR correlation coefficient (95% CI)	P value	CFVR correlation coefficient (95% CI)	P value
Dominant Systolic Forward travelling Compression Wave **(sFCW)**	0.71 (0.34,0.89)	0.002	0.59 (0.13,0.84)	0.014
Dominant early Diastolic Backward travelling Expansion Wave **(dBEW)**	-0.70 (-0.88,-0.31)	0.003	0.73 (-0.90,-0.38)	0.001
% Change sFCW cumulative intensity with PCI	-0.81 (-0.93,-0.52)	<0.001	-0.63 (-0.86,-0.20)	0.007
% Change dBEW cumulative intensity with PCI	-0.69 (-0.88,-0.30)	0.003	-0.59 (-0.84,-0.14)	0.012

### Recovery of accelerative wave intensity following PCI

**Fig 3:**
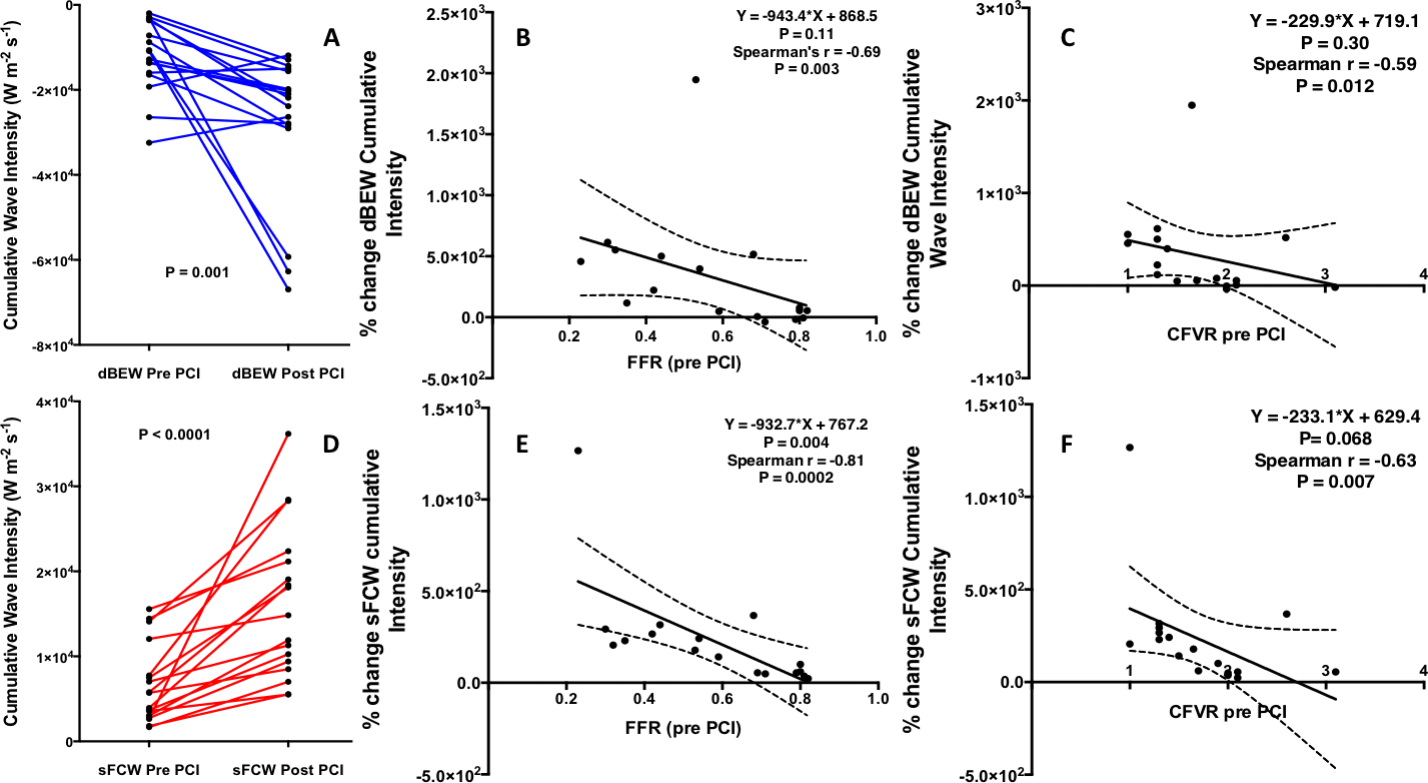
Effect of PCI on accelerative wave intensity and relationship with pre-PCI FFR and CFVR. **(A)** Cumulative intensity of the dBEW measured during hyperemia before and after PCI, **(B)** Percentage increase in dBEW intensity and pre-PCI FFR. PCI to lesions with the lowest baseline FFR values resulted in the largest percentage increase in diastolic suction wave intensity, whereas PCI to lesions with an FFR of close to 0.80 resulted in minimal increases. **(C)** Percentage increase in dBEW intensity and pre-PCI CFVR, **(D)** Cumulative intensity of the sFCW measured during hyperemia before and after PCI. **(E)** Percentage increase in sFCW intensity and pre-PCI FFR. PCI to lesions with the lowest baseline FFR values resulted in the largest percentage increase in sFCW intensity, whereas PCI to lesions with an FFR of close to 0.80 resulted in minimal increases. **(F)** Percentage increase in the sFCW intensity and pre-PCI CFVR. FFR, Fractional Flow reserve; CFVR, Coronary Flow Velocity Reserve; sFCW, systolic Forward travelling Compression Wave; dBEW, diastolic Backward travelling Expansion Wave.

PCI resulted in a median 117% (interquartile range 27–508) (P < 0.001) increase in dBEW intensity and a median 178% (interquartile range 55–280) (P < 0.0001) increase in sFCW (Fig 3A and 3D). Heterogeneity in this response to PCI was apparent, with treatment of some lesions associated with large increases in wave intensity.

The percentage increase in cumulative intensity of the dBEW following PCI correlated with baseline FFR (R = -0.69, P = 0.003) and with CFVR (R = -0.59, P = 0.012) (Table 5, Fig 3B and 3C). Additionally, the percentage increase in cumulative intensity of sFCW was correlated with both FFR and CFVR (R = -0.81, P<0.001 and R = -0.63, P = 0.007 respectively) (Table 5, Fig 3E and 3F). Consequently, larger relative increases in peak wave intensity followed PCI of lesions associated with greater baseline ischemia (lower FFR or CFVR). PCI of lesions with a baseline FFR < 0.79 resulted in a median 427% increase in dBEW cumulative intensity (range –38% to 1947%) whereas PCI of lesions with a baseline FFR > 0.79 resulted in a median 55% increase in dBEW cumulative intensity (range -19% to 77%) (P = 0.04). Similar findings were noted with the sFCW (Fig 3E and 3F). No correlations were noted between FFR or CFVR performed following PCI and the cumulative intensities of the 6 identified coronary waves (Table E in the S1 File).

### Procedural outcome

Whilst baseline cardiac troponin I (cTnI) was elevated (> 0.05 ng/mL) in 1 of the 17 patients at baseline, further cTnI elevations were noted in 15 of the 17 patients following the PCI procedure (P < 0.001). In 10 of these, cTnI was elevated to > 3 times the upper limit of the normal range.

### Reproducibility of CWIA measurements

Reproducibility of cumulative coronary wave intensity estimates was determined in 6 patients by repeating intracoronary adenosine administration pre and post PCI. The mean difference between replicate measurements of baseline sFCW intensity was 68 ± 39 10^3^ W m^-2^ s^-1^, and 83 ± 35 10^3^ W m^-2^ s^-1^ for the dBEW. The mean difference between replicate measurements of sFCW intensity following PCI was 96 ± 29 10^3^ W m^-2^ s^-1^, and 103 ± 38 10^3^ W m^-2^ s^-1^ for the dBEW. The coefficient of repeatability (CR), which defines the value below which 95% of the absolute differences between the two measurements lie [19, 20], was 153 10^3^ W m^-2^ s^-1^ for sFCW cumulative intensity (3.42% of mean sFCW intensity) and 178 10^3^ W m^-2^ s^-1^ for dBEW cumulative intensity (2.76% of mean dBEW intensity) measured pre PCI. Similarly, the CR was 200 10^3^ W m^-2^ s^-1^ for sFCW cumulative intensity (2.06% of mean sFCW intensity) and 216 10^3^ W m^-2^ s^-1^ for dBEW cumulative intensity (1.27% of mean dBEW intensity) measured post PCI.

## Discussion

This data, to our knowledge, is the first to characterize an association between FFR, CFVR and the cumulative intensity of the major accelerative coronary waves in the setting of an epicardial stenosis. Our principal finding that the cumulative intensities of both the sFCW and the dBEW were reduced proportionate to the reduction in FFR and CFVR measured prior to PCI suggests that abnormalities in generation or transmission of aortic pressure rise in systole and microvascular recoil in diastole may both contribute to the reduction in CBF observed with an epicardial stenosis. These findings are consistent with previous studies demonstrating progressive and profound reductions in diastolic flow with increasingly severe epicardial coronary stenoses and reduced cumulative intensity of the dBEW and CBF abnormalities in left ventricular hypertrophy (where early myocardial diastolic relaxation may be impaired) [1, 21, 22].

### Accelerative wave intensity following PCI

Target lesion PCI was associated with significant increases in the cumulative intensity of both major accelerative waves and the magnitude of this increase was directly proportional to FFR measured prior to PCI. Stenting of lesions with a baseline FFR of 0.80 or above did not result in significant changes in wave intensity measured following the procedure. As a FFR cutoff of 0.80 has been found to optimally discriminate between lesions associated with perfusion abnormalities on perfusion / stress imaging and those that are not, this finding suggests that impairment of accelerative wave intensity may play a role in the pathogenesis of the CBF defect associated an epicardial stenosis [6].

FFR was also closely correlated with the intensities of both accelerative waves calculated from the net coronary wave intensity profile (Single point wave speed independent and non-separated CWIA). The close correlations between peak net forward and backward travelling wave intensities with the cumulative intensities of the sFCW and the dBEW also suggest that changes in these waves underlie changes in net wave intensity. Whilst the close agreement of results obtained with peak net and separated wave intensity analysis cannot be seen as validation of the single point technique to estimate coronary wave speed, these findings further support the hypothesis that accelerative wave intensity is associated with the reduction of CBF observed in the setting of an epicardial stenosis.

### Pathogenesis of impaired diastolic suction wave intensity

Previous studies have suggested that early diastolic relaxation (with rapid relief of intramyocardial vascular compression) is the main determinant of dBEW intensity [1, 9]. Therefore reduced dBEW intensity may reflect either a reduction in transmission of pressure and flow due to lesion resistance or a quantitative reduction in the rate of early diastolic myocardial relaxation in the setting of myocardial ischaemia. Additionally, as wave intensity estimation is highly dependent on incremental changes in pressure and flow velocity with respect to time, a reduction in the amplitude of pressure and flow transmission distal to the stenosis may lead to the reductions in observed wave intensity. However, significant increases in cumulative intensity were only noted for the sFCW and dBEW following PCI and not all identified waves, as may be expected if a nonspecific diminution of wave intensity was responsible for the changes seen. Alternatively, the relative underfilling of the coronary circulation distal to an epicardial stenosis may contribute to the diminution of accelerative wave intensity in addition to decreased myocardial lusitropic function. In the most severe lesions (associated with FFR values less than 0.5), the reduction in CBF may lead to myocardial ischaemia at rest, reflected as impaired microvascular decompression in early diastole. Whilst left ventricular systolic contraction was noted to be normal by left ventriculography in 14 out of the 17 included patients, we cannot discount the possibility of subtle abnormalities of regional systolic and diastolic function as a result of myocardial ischaemia. The induction of hyperemia with adenosine may also precipitate myocardial ischaemia in the setting of a critical stenosis due to coronary steal or when a large translational pressure drop is generated [23, 24]. However, in less critical lesions myocardial ischemia is typically not induced as the rise in myocardial blood flow induced by adenosine is greater than the increase in myocardial oxygen consumption.

Whilst distal pressure and flow velocity as measured by pressure / flow wires are components of both the FFR and coronary wave intensity calculations, they differ by virtue of the dependence of FFR on proximal aortic pressure and CFVR on resting blood flow velocity. Neither proximal aortic pressure nor resting blood flow velocity are components of separated wave intensity. Similarly, coronary wave intensity analysis is dependent on incremental changes in distal flow velocity and pressure relative to time and not the mean absolute values of either parameter (which determine FFR and CFVR). Therefore, whilst some correlation between hyperaemic indices of coronary flow, (including FFR and CFVR) with hyperaemic coronary wave intensity, may be expected, the correlations that we have observed are not necessarily implicit.

In a study performed in patients with normal epicardial coronary arteries and severe aortic stenosis, an uncoupling of aortic pressure with the dBEW was proposed to account for the progressive falls in decompression wave intensity with pacing [11]. Abolition of the aortic outflow gradient with aortic valve replacement resulted in reversal of this response with progressive increases in intensity of the suction wave. In an analogous fashion, an epicardial stenosis may impair antegrade transmission of aortic diastolic pressure, thereby attenuating suction wave intensity. By restoring the vessel lumen, PCI may improve coupling of early diastolic relaxation with proximal diastolic pressure thereby contributing to increases in diastolic suction wave intensity following PCI.

### Study Limitations

Previous studies have questioned the validity of the single point wave speed method, as it yields estimates of coronary wave speed that are inconsistent with wave speed in large arteries [17, 18]. Recent studies have demonstrated that wave speed estimated with the single point method is consistently lower than directly measured wave speed following the administration of intravenous adenosine [16]. However single point wave speed estimates calculated during resting conditions have been shown to closely correspond to directly measured wave speed and were consequently used in our study. The close correlations between cumulative wave intensity obtained with this method and peak net wave intensity (which is independent of wave speed) further supports this approach. The peak amplitudes of net forward travelling compression and backward travelling decompression wave intensity were also strongly correlated with FFR and CFVR.

The dose of 24ug for the left coronary system and 18ug for the right coronary system is in keeping with doses used in large prospective studies that have validated the clinical use of FFR but has more recently been shown to result in underestimation of true FFR in a proportion of patients [5, 25]. Intracoronary delivery also results in a shorter duration of hyperemia, when compared to a continuous venous infusion. However, as the same intracoronary dose was used both prior to and following PCI, however, it is unlikely that a failure to achieve hyperemia would have systematically biased our results.

Both CFR and CFVR are sensitive to changes in baseline hemodynamic state, giving rise to alterations in basal flow. Whilst care was taken to perform measurements during periods of stable heart rate and systemic blood pressure, we cannot exclude variability in repeated CFVR measurements due to transient hemodynamic disturbances. Conversely, FFR measurement is considered to be substantially less sensitive to alterations in hemodynamic state

A relatively high rate of post-procedural troponin elevation was noted in this study, potentially confounding any association between post-PCI cumulative wave intensity and FFR / CFVR. The use of cTnI is known to increase the diagnosis of periprocedural infarction, compared to creatine kinase-myocardial band (CKMB) estimation [26]. Distal embolization of atheromatous material, female gender, diabetes, hypertension, lesion length > 20mm, renal dysfunction, multi-vessel disease and left anterior descending coronary artery disease may have also contributed to the incidence of peri-procedural MI [27]. This may partially account for the lack of association between post-PCI dBEW cumulative intensity and FFR / CFVR.

Left ventriculography was used to approximate left ventricular function and echocardiography was not uniformly performed in our study. Non-invasive echocardiography with measurement of ventricular wall thickness or the application of tissue Doppler imaging may have provided useful correlation of changes in diastolic performance with the early diastolic suction wave. Estimation of myocardial diastolic function with a validated index such as the left ventricular diastolic time constant may have provided further mechanistic insight into the recovery of diastolic suction wave intensity following PCI [28]. However both approaches are relatively insensitive to changes in segmental diastolic function, which is pertinent to the evaluation of diastolic dysfunction in the territory of the interrogated coronary artery.

Finally, despite being a highly lesion specific index of ischaemia, FFR also reflects myocardial viability in the territory perfused by the stenosed vessel[4, 6, 29, 30]. Loss of viability in the perfused territory leads to increases in measured FFR, reflecting a reduction in hyperemic flow [4, 29–31]. In this study, patients with known previous infarction in the territory of the target vessel were excluded. Consequently, the correlation between dBEW intensity and FFR likely reflects impaired diastolic microvascular decompression in viable myocardium only. Similarly, the association between CFVR (a less lesion specific measure of coronary flow) and suction wave intensity may not be applicable in those patients with a substantial proportion of non-viable myocardium.

## Conclusions

In this study, we have shown strong associations between FFR, CFVR and the intensity of both major accelerative waves. Impairment of dBEW intensity represents a hitherto unrecognized mechanism driving abnormal hyperemic flow in the setting of a coronary stenosis. Revascularization with PCI resulted in significant increases in the cumulative intensity of both the sFCW and the dBEW. Taken together, these findings provide further insights into the pathogenesis of coronary ischaemia and into the basic mechanisms through which epicardial coronary revascularization improves myocardial blood flow.

## Supporting Information

S1 File Haemodynamic and PCI characteristics of the coronary arteries and lesions studied. Data are presented as mean (SD) for continuous variables. BMI, Body Mass index; Hb, Hemoglobin; SBP, Systolic Blood pressure; DBP, Diastolic Blood pressure; HR, Heart rate; LAD, Left Anterior Descending coronary artery; LCx, Left Circumflex coronary artery; OM1, First Obtuse Marginal branch; RCA, Right Coronary Artery. 3All lesions were graded according to the AHA lesion classification system [1]. 1, Bare Metal stent; 2, Drug eluting stent. Prox, proximal; Mid, mid segment (**Table A**). Individual participant hemodynamic and peri-procedural characteristics. AMI, Acute myocardial infarction; RCA, Right coronary artery; LAD, Left anterior descending coronary artery; LCx, Left circumflex coronary artery; cTnI, Cardiac Troponin I; FFR, Fractional Flow reserve; CFVR, Coronary Flow Velocity Reserve; EST, Exercise stress test; MPI, Sestamibi Myocardial Perfusion scan (**Table B**). Univariate linear regression analyses of FFR and CFVR with baseline haemodynamic and patient variables. HR, Heart Rate; SBP, Systolic Blood Pressure; DBP, Diastolic Blood Pressure; PP, Pulse Pressure; FFR, Fractional Flow reserve; CFVR, Coronary Flow Velocity Reserve; sFCW, systolic Forward travelling Compression Wave; dBEW, diastolic Backward travelling Expansion Wave (**Table C**). Univariate linear regression analyses of baseline (pre-PCI) sFCW and dBEW cumulative intensity with baseline haemodynamic and patient variables. HR, Heart Rate; SBP, Systolic Blood Pressure; DBP, Diastolic Blood Pressure; PP, Pulse Pressure; FFR, Fractional Flow reserve; CFVR, Coronary Flow Velocity Reserve; sFCW, systolic Forward travelling Compression Wave; dBEW, diastolic Backward travelling Expansion Wave (**Table D**). Correlations between FFR / CFVR measured distal to the stented lesion post PCI with the cumulative intensities of the six coronary waves. No significant correlations were observed between FFR / CFVR and cumulative accelerative wave intensity post PCI. The Spearman correlation coefficient was applied to estimate statistical significance. FFR, Fractional Flow reserve; CFVR, Coronary Flow Velocity Reserve; sFCW, systolic Forward travelling Compression Wave; dBEW, diastolic Backward travelling Expansion Wave (**Table E**).
(DOCX)

## Abbreviations

CFVR: Coronary Flow Velocity Reserve
CBF: Coronary Blood Flow
CWIA: Coronary Wave Intensity Analysis
PCI: Percutaneous Coronary Intervention
CABG: Coronary Artery Bypass Grafting
CK-MB: Creatine Kinase Myocardial band
dBEW: Diastolic Backward travelling Expansion Wave
FFR: Fractional Flow Reserve
sFCW: Systolic Forward travelling Compression Wave
cTnI: cardiac Troponin I
