# Supplementary material for: Percutaneous Coronary Intervention Enhances Accelerative Wave Intensity in Coronary Arteries
Source: PLoS One. 2015 Dec 11;10(12):e0142998. doi: 10.1371/journal.pone.0142998 (PMC4676634; doi:10.1371/journal.pone.0142998)
Supplement: S1 File — Data are presented as mean (SD) for continuous variables. BMI, Body Mass index; Hb, Hemoglobin; SBP, Systolic Blood pressure; DBP, Diastolic Blood pressure; HR, Heart rate; LAD, Left Anterior Descending coronary artery; LCx, Left Circumflex coronary artery; OM1, First Obtuse Marginal branch; RCA, Right Coronary Artery. 3All lesions were graded according to the AHA lesion classification system [1]. 1, Bare Metal stent; 2, Drug eluting stent. Prox, proximal; Mid, mid segment (Table A). Individual participant hemodynamic and peri-procedural characteristics. AMI, Acute myocardial infarction; RCA, Right coronary artery; LAD, Left anterior descending coronary artery; LCx, Left circumflex coronary artery; cTnI, Cardiac Troponin I; FFR, Fractional Flow reserve; CFVR, Coronary Flow Velocity Reserve; EST, Exercise stress test; MPI, Sestamibi Myocardial Perfusion scan (Table B). Univariate linear regression analyses of FFR and CFVR with baseline haemodynamic and patient variables. HR, Heart Rate; SBP, Systolic Blood Pressure; DBP, Diastolic Blood Pressure; PP, Pulse Pressure; FFR, Fractional Flow reserve; CFVR, Coronary Flow Velocity Reserve; sFCW, systolic Forward travelling Compression Wave; dBEW, diastolic Backward travelling Expansion Wave (Table C). Univariate linear regression analyses of baseline (pre-PCI) sFCW and dBEW cumulative intensity with baseline haemodynamic and patient variables. HR, Heart Rate; SBP, Systolic Blood Pressure; DBP, Diastolic Blood Pressure; PP, Pulse Pressure; FFR, Fractional Flow reserve; CFVR, Coronary Flow Velocity Reserve; sFCW, systolic Forward travelling Compression Wave; dBEW, diastolic Backward travelling Expansion Wave (Table D). Correlations between FFR / CFVR measured distal to the stented lesion post PCI with the cumulative intensities of the six coronary waves. No significant correlations were observed between FFR / CFVR and cumulative accelerative wave intensity post PCI. The Spearman correlation coefficient was applied to estimate [file pone.0142998.s001.docx]

**Supplementary Information**

**Table A: Baseline Hemodynamic and PCI characteristics**

| Patient number | Gender | Age | BMI | Hb | SBP | DBP | HR beats per min | Stenosed artery segment | Vessel Diameter (mm) | Aha lesion classification^3^ | % diameter stenosis | Type of stent | Stent Dimensions (mm) |
| --- | --- | --- | --- | --- | --- | --- | --- | --- | --- | --- | --- | --- | --- |
| 1 | Male | 62 | 31.6 | 164 | 97.1 (5.8) | 65.4 (3.7) | 61 | Mid LAD | 3.3 | B | 70 | Lekton^1^ | 3x15 |
| 2 | Male | 66 | 28.9 | 140 | 109.0 (12.8) | 58.8 (8.2) | 52 | Prox LAD | 2.7 | C | 50 | R stent^1^ | 4.0x28 |
| 3 | Female | 71 | 31.2 | 126 | 119.0(10.6) | 53.2 (2.6) | 58 | Prox LCx | 3.8 | B | 74 | S7 stent^1^ | 3.0 x24 |
| 4 | Male | 61 | 28.4 | 140 | 146.6 (5.6) | 78.6 (4.5) | 49 | Prox OM1 | 2.2 | B | 80 | S660^1^ | 2.5x24 |
| 5 | Female | 54 | 24.2 | 136 | 121.5 (4.0) | 64.2 (4.4) | 53 | Mid LAD | 2.6 | B | 37 | S660^1^ | 2x12 |
| 6 | Female | 73 | 19.7 | 112 | 111.2 (7.6) | 62.3 (3.9) | 47 | Prox RCA | 2.5 | B | 56 | S7^1^ | 3.0x12 |
| 7 | Male | 62 | 25.2 | 147 | 108.7 (7.1) | 61.5 (2.3) | 50 | Prox RCA | 3.4 | B | 90 | Express^1^ | 4.5x20 |
| 8 | Male | 71 | 25.4 | 148 | 105.6 (6.5) | 55.2 (7.5) | 51 | Mid LAD | 4.0 | B | 86 | Driver^1^ | 3x15 |
| 9 | Female | 83 | 25.3 | 121 | 156.1 (6.6) | 68.9 (5.7) | 54 | Mid LAD | 2.6 | B | 84 | Driver^1^ | 3x12 |
| 10 | Female | 69 | 20.4 | 147 | 142.8 (16.3) | 69.2 (6.5) | 69 | Mid LCx | 2.5 | B | 82 | Tsunami Gold^1^ | 3x20 |
| 11 | Female | 75 | 22.7 | 134 | 135.5 (12.7) | 62.0 (3.8) | 51 | Mid LAD | 3.2 | A | 63 | S660^1^ | 2.5x9 |
| 12 | Male | 51 | 30.3 | 124 | 151.4 (5.8) | 86.5 (2.0) | 62 | Mid RCA | 2.2 | C | 73 | Express^1^ | 2.75x16 |
| 13 | Female | 46 | 24.1 | 131 | 104.3 (6.8) | 61.0 (2.7) | 80 | Mid RCA | 2.8 | B | 75 | Driver | 3.0x24 |
| 14 | Male | 65 | 21.9 | 125 | 176.3 (6.8) | 78.7 (2.6) | 65 | Prox-Mid LAD | 2.4 | A | 68 | Taxus ^2^ | 2.5x16 |
| 15 | Male | 42 | 30.9 | 156 | 119.3 (4.4) | 72.5 (1.9) | 64 | Prox-Mid LAD | 2.3 | B | 95 | Taxus ^2^ | 3 x 32 |
| 16 | Female | 73 | 25.8 | 138 | 115.0 (7.0) | 65.3 (3.6) | 50 | Mid OM1 | 2.6 | B | 64 | Express^1^ | 2.25x13 |
| 17 | Male | 64 | 23.6 | 153 | 123.5 (6.0) | 76.4 (3.2) | 64 | Prox LAD | 2.2 | C | 85 | Taxus ^2^ | 3x28 |

**Table A:** Haemodynamic and PCI characteristics of the coronary arteries and lesions studied. Data are presented as mean (SD) for continuous variables. BMI, Body Mass index; Hb, Hemoglobin; SBP, Systolic Blood pressure; DBP, Diastolic Blood pressure; HR, Heart rate; LAD, Left Anterior Descending coronary artery; LCx, Left Circumflex coronary artery; OM1, First Obtuse Marginal branch; RCA, Right Coronary Artery. ^3^All lesions were graded according to the AHA lesion classification system [1] . ^1^, Bare Metal stent; ^2^, Drug eluting stent. Prox, proximal; Mid, mid segment.

**Table B: Additional baseline and peri-procedural characteristics**

| Patient number | Previous AMI | Days since AMI | Territory | Infarct artery | Functional testing | | Pre PCI FFR | | Post PCI FFR | Pre PCI CFVR | Post PCI CFVR | Pre procedural cTnI | | Post Procedural cTnI |  |
| --- | --- | --- | --- | --- | --- | --- | --- | --- | --- | --- | --- | --- | --- | --- | --- |
| 1 | Yes | 104 | Inferior | RCA |  | 0.69 | | 0.90 | | 2.1 | 2.7 | 0.04 | 0.51 | | |
| 2 | No |  |  |  | EST | 0.59 | | 0.91 | | 1.5 | 1.9 | 0.04 | 6.11 | | |
| 3 | No |  |  |  |  | 0.42 | | 0.98 | | 1.3 | 3.8 | 0.04 | 0.20 | | |
| 4 | Yes | 2207 | Inferior | RCA | EST | 0.44 | | 0.98 | | 1.3 | 2.5 | 0.04 | 0.46 | | |
| 5 | No |  |  |  | MPI | 0.71 | | 0.81 | | 2.0 | 3.0 | 0.04 | 0.51 | | |
| 6 | Yes | 135 | Antero-septal | LAD |  | 0.82 | | 0.99 | | 2.0 | 4.7 | 0.04 | 0.05 | | |
| 7 | No |  |  |  |  | 0.35 | | 0.98 | | 1.0 | 3.8 | 0.05 | 0.28 | | |
| 8 | Yes | 65 | Infero-lateral | LCx |  | 0.68 | | 0.82 | | 2.6 | 3.3 | 0.04 | 0.57 | | |
| 9 | No |  |  |  |  | 0.82 | | 0.93 | | 2.1 | 2.3 | 0.04 | 0.07 | | |
| 10 | Yes | 149 | Infero-posterior | RCA |  | 0.30 | | 0.94 | | 1.3 | 2.3 | 0.05 | 0.38 | | |
| 11 | No |  |  |  |  | 0.79 | | 0.85 | | 3.1 | 3.8 | 0.04 | 0.26 | | |
| 12 | No |  |  |  | MPI | 0.35 | | 0.81 | | 1.3 | 3.3 | 0.06 | 0.04 | | |
| 13 | Yes | 245 | Inferior | RCA | MPI | 0.54 | | 0.95 | | 1.4 | 2.6 | 0.04 | 0.07 | | |
| 14 | No |  |  |  | EST | 0.80 | | 0.90 | | 1.9 | 1.7 | 0.04 | 0.19 | | |
| 15 | No |  |  |  |  | 0.20 | | 0.90 | | 1.0 | 3.2 | 0.04 | 0.31 | | |
| 16 | No |  |  |  |  | 0.80 | | 1.00 | | 1.7 | 3.3 | 0.04 | 0.27 | | |
| 17 | No |  |  |  | MPI | 0.50 | | 0.90 | | 1.7 | 2.5 | 0.04 | 2.52 | | |

**Table B:** Individual participant hemodynamic and peri-procedural characteristics. AMI, Acute myocardial infarction; RCA, Right coronary artery; LAD, Left anterior descending coronary artery; LCx, Left circumflex coronary artery; cTnI, Cardiac Troponin I; FFR, Fractional Flow reserve; CFVR, Coronary Flow Velocity Reserve; EST, Exercise stress test; MPI, Sestamibi Myocardial Perfusion scan.

**Table C: Univariate Linear Regression analyses – FFR and CFVR**

|  |  | FFR | |  | CFVR | |
| --- | --- | --- | --- | --- | --- | --- |
| **Variables** | **Regression Coefficient** | **Univariate R^2^** | **Univariate p value** | **Regression Coefficient** | **Univariate R^2^** | **Univariate p value** |
| Age | **30.5 (11.2)** | **0.330** | **0.016** | **10.3 (4.2)** | **0.284** | **0.028** |
| HR | -14.9 (10.3) | 0.123 | 0.168 | -5.1 (3.8) | 0.110 | 0.194 |
| SBP | 6.1 (27.7) | 0.003 | 0.830 | 0.8 (10.0) | 0.0004 | 0.935 |
| DBP | -9.9 (11.2) | 0.049 | 0.392 | -4.4 (4.0) | 0.075 | 0.287 |
| Haemoglobin | -26.1 (16.6) | 0.143 | 0.135 | -3.1 (6.3) | 0.016 | 0.624 |
| PP | 15.9 (21.2) | 0.036 | 0.465 | 5.2 (7.7) | 0.030 | 0.508 |
| Stenosis severity (%) | **21.0 (18.5)** | **0.298** | **0.023** | 5.3 (6.8) | 0.117 | 0.180 |
| sFCW intensity | **16146 (4187)** | **0.498** | **0.002** | **4737 (1752)** | **0.328** | **0.016** |
| dBEW intensity | **-27594 (8162)** | **0.432** | **0.004** | **-11624 (2519)** | **0.587** | **0.0003** |

**Table C:** Univariate linear regression analyses of FFR and CFVR with baseline haemodynamic and patient variables. HR, Heart Rate; SBP, Systolic Blood Pressure; DBP, Diastolic Blood Pressure; PP, Pulse Pressure; FFR, Fractional Flow reserve; CFVR, Coronary Flow Velocity Reserve; sFCW, systolic Forward travelling Compression Wave; dBEW, diastolic Backward travelling Expansion Wave.

**Table D: Univariate Linear Regression analyses – sFCW and dBEW cumulative intensity**

|  | sFCW | | | dBEW | | | |
| --- | --- | --- | --- | --- | --- | --- | --- |
| **Variables** | **Regression Coefficient** | **Univariate R^2^** | **Univariate p value** | **Regression Coefficient** | **Univariate R^2^** | **Univariate p value** |  |
| **Age** | **1.33 (0.50)** | **0.329** | **0.016** | -0.36 (0.31) | 0.082 | 0.264 |  |
| HR | -0.55 (0.46) | 0.088 | 0.248 | 0.30 (0.25) | 0.087 | 0.252 |  |
| SBP | 1.79 (1.12) | 0.145 | 0.132 | 0.10 (0.66) | 0.018 | 0.895 |  |
| DBP | -0.14 (0.50) | 0.005 | 0.780 | 0.22 (0.27) | 0.045 | 0.415 |  |
| **PP** | **1.93 (0.80)** | **0.277** | **0.030** | -0.14 (0.51) | 0.005 | 0.796 |  |
| Stenosis severity (%) | -1.45 (0.75) | 0.199 | 0.073 | -0.29 (0.45) | 0.026 | 0.024 |  |
| **Haemoglobin** | **-2.11 (0.54)** | **0.508** | **0.0013** | 0.07 (0.42) | 0.002 | 0.876 |  |

**Table D:** Univariate linear regression analyses of baseline (pre-PCI) sFCW and dBEW cumulative intensity with baseline haemodynamic and patient variables. HR, Heart Rate; SBP, Systolic Blood Pressure; DBP, Diastolic Blood Pressure; PP, Pulse Pressure; FFR, Fractional Flow reserve; CFVR, Coronary Flow Velocity Reserve; sFCW, systolic Forward travelling Compression Wave; dBEW, diastolic Backward travelling Expansion Wave.

**Table E: Correlations between post PCI cumulative accelerative wave intensity and FFR / CFVR**

|  | Post PCI  FFR correlation coefficient (95% CI) | P value | Post PCI  CFVR correlation coefficient (95% CI) | P value |
| --- | --- | --- | --- | --- |
| . Early Systolic Backward travelling Compression Wave | 0.13 (-0.39,0.59) | 0.61 | 0.30 (-0.22,0.69) | 0.24 |
| 2. Dominant Systolic Forward travelling Compression Wave  (sFCW) | -0.18 (-0.62,0.34) | 0.47 | 0.18 (-0.34,0.62) | 0.49 |
| 3. Late Systolic Backward travelling Compression Wave | 0.22 (-0.31,0.64) | 0.40 | -0.32 (-0.70,0.21) | 0.21 |
| 4. Early Diastolic Forward travelling Expansion Wave | 0.01 (-0.48,0.50) | 0.96 | 0.05 (-0.45,0.53) | 0.84 |
| 5. Dominant early Diastolic Backward travelling Expansion Wave (dBEW) | 0.21 (-0.32,-0.64) | 0.42 | 0.16 (-0.36,0.61) | 0.52 |
| 6. Late diastolic forward travelling pushing wave | 0.04 (-0.46,0.52) | 0.89 | 0.28 (-0.25,0.68) | 0.28 |

**Table E:** Correlations between FFR / CFVR measured distal to the stented lesion post PCI with the cumulative intensities of the six coronary waves. No significant correlations were observed between FFR / CFVR and cumulative accelerative wave intensity post PCI. The Spearman correlation coefficient was applied to estimate statistical significance. FFR, Fractional Flow reserve; CFVR, Coronary Flow Velocity Reserve; sFCW, systolic Forward travelling Compression Wave; dBEW, diastolic Backward travelling Expansion Wave.

**References:**

1. *Guidelines for percutaneous transluminal coronary angioplasty. A report of the American College of Cardiology/American Heart Association Task Force on Assessment of Diagnostic and Therapeutic Cardiovascular Procedures (Committee on Percutaneous Transluminal Coronary Angioplasty).* J Am Coll Cardiol, 1993. **22**(7): p. 2033-54.
